# Supplementary material for: Analysis of PPARγ Signaling Activity in Psoriasis
Source: Int J Mol Sci. 2021 Aug 10;22(16):8603. doi: 10.3390/ijms22168603 (PMC8395241; doi:10.3390/ijms22168603)
Supplement: Supplementary file 1 [file ijms-22-08603-s001.zip › Supplemental materials_Analysis of PPARg signaling activity in psoriasis/Pathway models/Models images and html files/PPARG inhibits phenotypes assotiated with psoriasis/237503.html]

sarcoidosis


# Disease sarcoidosis

|  |  |
| --- | --- |
| URN | urn:agi-meshdis:Sarcoidosis |
| Total Entities | 8 |
| Connectivity | 2461 |
| Name | sarcoidosis |

---

|  |  |
| --- | --- |
| ChildConcepts | Lofgren's syndrome |
|  | ocular sarcoidosis |
|  | skin sarcoidosis |
|  | cardiac sarcoidosis |
|  | neurosarcoidosis |
|  | Heerfordt's syndrome |
|  | pulmonary sarcoidosis |
|  | Jungling syndrome |

---

|  |  |
| --- | --- |
| Pathway | PPARG inhibits phenotypes assotiated with psoriasis |

---

|  |  |
| --- | --- |
| MedScan ID | 9010670 |

---

|  |  |
| --- | --- |
| Alias | Schaumanns disease |
|  | Besnier-Boeck-Schaumann's syndromes |
|  | Besnier-Boeck-Schaumann diseases |
|  | Boeck diseases |
|  | Darier-Roussy sarcoid |
|  | Boeck disease |
|  | Besnier-Boeck-Schaumann disease |
|  | Schaumann's disease |
|  | Schaumann's diseases |
|  | benign lymphogranulomatosis |
|  | Hutchinson-Boeck diseases |
|  | Besnier-Boeck disease |
|  | benign lymphogranulomatoses |
|  | Besnier-Boeck |
|  | Hutchinson-Boeck sarcoid |
|  | sarcoids of Boeck |
|  | Boeck's diseases |
|  | Besnier-Boeck-Schaumanns diseases |
|  | sarcoids |
|  | Besnier-Boeck diseases |
|  | Boeck sarcoids |
|  | Besnier-Boeck-Schaumann's disease |
|  | Besnier-Boeck syndrome |
|  | Schaumann disease |
|  | Besnier-Boeck-Schaumann's syndrome |
|  | Besnier-Boeck-Schaumann's diseases |
|  | Besnier-Boeck-Schaumanns syndromes |
|  | sarcoidosis |
|  | Schaumann diseases |
|  | lymphogranuloma benignum |
|  | Boeck's sarcoid |
|  | sarcoid, Boeck's |
|  | sarcoid of Boeck |
|  | Besnier-Boeck syndromes |
|  | Boeck's sarcoids |
|  | Hutchinson-Boeck |
|  | Boeck's disease |
|  | Hutchinson-Boeck disease |
|  | Boeck's sarcoidosis |
|  | Hutchinson-Boeck granulomatosis |
|  | Boeck's sarcoidoses |
|  | Besnier-Boeck-Schaumanns syndrome |
|  | Boeck sarcoid |
|  | Hutchinson-Boeck sarcoids |
|  | Darier-Roussy sarcoids |
|  | Besnier-Boeck-Schaumann syndromes |
|  | Besnier-Boeck-Schaumann syndrome |
|  | Besnier-Boeck-Schaumanns disease |
|  | Hutchinson-Boeck granulomatoses |
|  | benign lymphogranulomatosis of Schaumann |
|  | sarcoidoses |
|  | sarcoid |
|  | jungling syndrome |
|  | Lupus Pernio |
|  | Disease, Besnier-Boeck |
|  | jungling syndromes |
|  | Diseases, Besnier-Boeck |
|  | neurosarcoidosis |
|  | Lupus Pernio, Besnier |

---

|  |  |
| --- | --- |
| MeSH ID | D012507 |
|  | M0019426 |

---

|  |  |
| --- | --- |
| MeSH Heading | Sarcoidosis |

---

|  |  |
| --- | --- |
| Orphanet ID | 797 |

---
